# Building a bridal chamber: development of the thalamus

**DOI:** 10.1016/j.tins.2010.05.003

**Published:** 2010-08

**Authors:** Steffen Scholpp, Andrew Lumsden

**Affiliations:** 1 Karlsruhe Institute of Technology (KIT), Institute for Toxicology and Genetics, 76021 Karlsruhe, Germany; 2 MRC Centre for Developmental Neurobiology, King's College London, London, SE1 1UL, UK

## Abstract

The thalamus is a central brain region that plays a crucial role in distributing incoming sensory information to appropriate regions of the cortex. The thalamus develops in the posterior part of the embryonic forebrain, where early cell fate decisions are controlled by a local signaling center – the mid-diencephalic organizer – which forms at the boundary between prospective prethalamus and thalamus. In this review we discuss recent observations of early thalamic development in zebrafish, chick, and mouse embryos, that reveal a conserved set of interactions between homeodomain transcription factors. These interactions position the organizer along the neuraxis. The most prominent of the organizer's signals, Sonic hedgehog, is necessary for conferring regional identity on the prethalamus and thalamus and for patterning their differentiation.

## The bridal chamber

The term ‘thalamus’ (from the Greek *thalamos*: a chamber) was used by Galen in *De Usu Partium* by way of comparing the human brain with the ground plan of a Greek house, with the bridal chamber at its heart – emphasizing the central role and location of the twinned bulb-shaped structures that form at the top of the brainstem on either side of the third ventricle [1]. The thalamic complex is located in the diencephalic (posterior) part of the forebrain and includes the prethalamus and thalamus (formerly known as ventral thalamus and dorsal thalamus, respectively). This complex is the major sensory relay station of the brain, receiving all inputs (except olfaction) and connecting reciprocally with the overlying cortex; therefore, in a more philosophical way, the thalamus has been described as ‘the gateway to consciousness’ [2]. The prethalamus and the thalamus are separated by the Zona Limitans Intrathalamica (ZLI), a narrow strip of cells that traverses the neural tube, marked by a ridge on the ventricular surface, that has been described morphologically in a wide variety of vertebrates [3].

## A local organizer of thalamic development

Patterning large and elaborate brain regions, such as the neocortex or the cerebellum, is known to require instructive cell populations – ‘local organizers’ – that are located at prominent morphological discontinuities or boundaries in the neural primordium and that establish concentration gradients of morphogenetic signal molecules in adjacent responsive tissues, thereby informing neighboring cells about their position and fate. Similarly in the thalamus, another complex brain structure, the ZLI expresses members of the Sonic hedgehog (Shh) signal molecule family, together with other secreted factors such as Wnts and fibroblast growth factors (FGFs), suggesting that it functions as a local organizer of thalamic development. Indeed, each of these factors has been implicated in local organizer functions elsewhere in the brain, such as the floor plate of the spinal cord and the midbrain–hindbrain boundary (MHB). The defining characteristics of an organizer include the prevention of mixing between its cells and neighbors, which stabilizes the composition and location of the organizer population and ensures stability of the morphogen gradient (Box 1,2). Previous work has shown that the ZLI is indeed cell-lineage restricted at its borders, with the prethalamus anteriorly and the thalamus posteriorly [4]. Another key character is the ability of an organizer to induce ectopic cell fates in host tissue following heterotopic transplantation. Again, grafting and explant experiments have shown that mid-diencephalic cells impose altered cell fate by the ectopic induction of thalamic and prethalamic genes [5,6]. Therefore, the mid-diencephalic ZLI has the characteristics of a local organizer.

In this review we summarize the molecular events leading to the formation of this organizing cell population, now referred to as the mid-diencephalic organizer (MDO), and show how its signals are crucial for development of the entire thalamic complex.

## Positioning and induction of the MDO: a Fez–Otx interface

Induction of the MDO has been proposed to result from interaction between prechordal (anteriormost) and epichordal regions of the neural plate, which meet in the mid-diencephalon. Grafting and co-culture experiments in chick have shown that the juxtaposition of diverse prechordal and epichordal neural tissues can induce Shh expression at the interface [5,6]. A genetic mechanism has been proposed for chick in which the apposition of the expression domains of the transcription factors Six3 (prechordal) and Irx3 (epichordal) would induce MDO formation [7]. However, a difficulty with this model is that in other vertebrates there is a gap between the *Six3* domain and that of orthologous *Irx* genes – *Irx1* in mouse [8] and both *irx1b* and *irx7* in zebrafish [9]. Furthermore, absence of either these factors should prevent MDO formation, yet this initiates correctly in *Six3* knock-out mice and in *Irx1b* knock-down fish [10–12].

**Figure 1:**
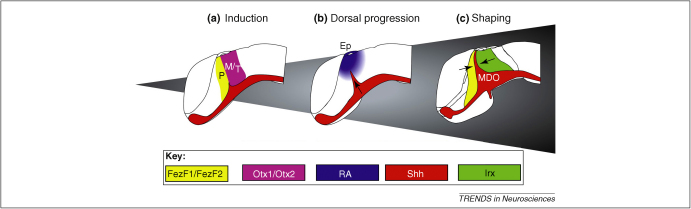
Development of the mid-diencephalic organizer. **(a)** The MDO is induced at the interface of the Fez-positive prethalamus anlage and the Otx-positive MDO/thalamus anlage. **(b)** Following induction, the principal signal of the MDO – Shh – is expressed from ventral to dorsal, possibly limited dorsally by RA signaling from the epithalamus. **(c)** Shh expression within the MDO is limited anteroposteriorly by the repressive function of neighboring transcription factors, Fez and Irx. Expression of Fez and Irx establish the prethalamic and thalamic anlagen as differentially competent fields, determining their subsequent response to Shh signaling from the MDO. Ep, epithalamus; MDO,mid-diencephalic organizer; M/T, anlage of the mid-diencephalic organizer and thalamus; P, prethalamic anlage.

Incorporating new findings from fish, chick, and mouse, we propose a new model where interaction between Fez and Otx transcription factor domains establishes the MDO (Figure 1A). *Fez* is expressed in the prechordal anlage from late gastrulation stages onwards [13,14], and functional experiments show that *Fez* is required for MDO induction in mice and fish [8,15]. We recently analyzed the function of *Otx* in regionalization of the zebrafish neural plate and found that the domains of *Otx1* and *Otx2* abut that of *Fez*. Furthermore, downregulation of Otx1/Otx2 immediately before MDO formation prevents MDO formation, showing a requirement for Otx function [11]. Our findings are consistent with the analysis of the *Otx1*^*−/−*^*/Otx2*^*+/−*^ mouse, which shows a similar lack of MDO formation [16]. In mouse, it appears that the FezF1/FezF2 expression domain abuts that of Irx1, suggesting that Irx1 determines the posterior border of the MDO [8]. However, in zebrafish the Irx1 orthologs *irx1b/irx7* are expressed more caudally [17], making a direct interface interaction between these factors unlikely. Instead, we propose a new model where Fez and Otx factors confront one another and form a sharp interface, possibly through mutual cross-repression.

## Onset of signal molecule expression

After correct antero-posterior (AP) positioning by a Fez/Otx-mediated inductive process, the most prominent signaling molecule of the MDO, Shh, is induced. The expression domain of Shh expands progressively from the Shh-positive basal plate towards the roof plate [4,18–20]. This ventral-to-dorsal progression, together with the fact that expression of Shh in the MDO is under the control of the same enhancer element as that for forebrain basal plate expression [21,22], suggests that the Shh-positive cells might migrate dorsally from the basal plate to contribute to the MDO. However, fate map analysis shows that few basal plate cells migrate dorsally [17,23], and that the MDO must therefore be recruited from alar plate cells. Vertical induction via signaling from the basal plate would be another explanation for the ventral-to-dorsal spread of Shh expression, but zebrafish mutants lacking the basal plate have a near-normal MDO, showing that induction of Shh in the MDO is independent of ventral Shh signaling, or indeed any ventral signaling [23,24]. These *in vivo* data are supported by explant studies of chick forebrain tissues showing that ventral tissue is dispensable for MDO induction and extension [6]. If neither cell movement nor signaling from the basal plate contribute to the dynamic Shh expression, what else could explain the ventral-to-dorsal progression of MDO maturation? One possibility is decaying repression from dorsal, rather than activation from ventral (Figure 1B) [6,20]. A candidate signal is retinoic acid synthesized by the cytochrome p450 family enzyme Cyp1B1, which is expressed in the diencephalic roof plate in chick at 30 somites and becomes reduced during late somitogenesis stages [25]. Indeed, addition of retinoic acid to the culture medium blocks Shh expression in diencephalic explants [6].

Following maturation, the MDO domain of Shh has a characteristic tapered shape. Because the domains of Fez and Irx are also separated by a wedge-shaped gap it is likely that Shh can only be induced in this Fez/Irx-free zone, which was previously identified as the prospective ZLI by its lack of lunatic fringe expression [4]. Indeed, broadening of the gap by single knock-down of FezF2 leads to anterior broadening of the MDO [15]. Similarly, Irx1b morphant embryos show an increase of the posterior extent of the MDO [11]. This suggests repressive functions for Fez and Irx in delineating the borders of the MDO (Figure 1C). Whereas the mechanism that prevents cell mixing at these borders [4,18] has yet to be elucidated, the precise coincidence of lineage restriction with the expression limits of FezF1/FezF2 and Irx1 transcription factors suggests that they exert upstream control on the process [26]. A further factor limiting the width of the organizer might be Pax6, because *pax6* null m*ice* show a broader expression of Shh [27].

## The MDO as an instructive signaling center

Hedgehog family genes are expressed in the MDO of all vertebrate model organisms examined, including lamprey [28], zebrafish [29], frog [30], chick [31] and mouse [32]. Although other secreted signals, principally FGFs and Wnts, are also expressed there [33], a significant organizing function is mediated by Shh, the predominant Hedgehog family member expressed in this region [5,23,34]. Experimental abrogation of Shh signaling in both chick and zebrafish results in the loss of genetic fate determinants and cell identity in both the prethalamus and the thalamus [23,34]. Furthermore, ectopic activation of the Shh pathway by misexpression of the signal transduction component of its receptor (smoothened, SmoM2) induces the expression of thalamic markers such as Gbx2, Neurog2, Olig2 and Olig3 in the mouse pretectum [35].

**Figure 2:**
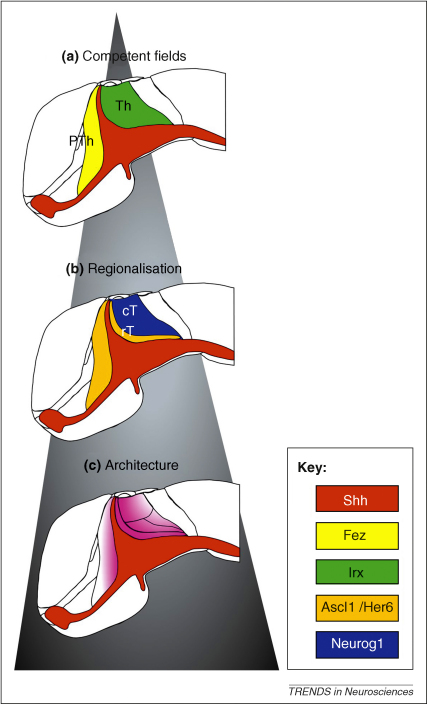
Three steps in regionalization of the thalamus. **(a)** The transcription factors Fez and Irx define properties anteriorly and posteriorly to the MDO. **(b)** The bHLH factor Her6 determines neuronal identity: in the Her6-positive domains, Shh induces Ascl1, and in the Her6-negative domain, Shh induces Neurogenin1 (Neurog1). **(c)** Shh (red) influences the sizes of the thalamic nuclei by a Shh protein concentration gradient (fading red). cT, caudal thalamus; PTh, prethalamus; rT, rostral thalamus; Th, thalamus.

We identify three Shh-dependent steps during patterning of the thalamic anlagen. First, the prethalamus and the thalamus acquire distinct identities following exposure to Shh – implying that the two diencephalic regions have different responsiveness, or competence (Figure 2A). This is due, at least in part, to the prior expression of Irx in the thalamic anlage: mis-expression of Irx3 in the prospective prethalamus leads to Shh-dependent ectopic expression of the thalamic markers Sox14 and Gbx2 [34]. Genes encoding Fez have been suggested to play a similar role in the prethalamus [8,15]. Initial AP polarity of the neural plate is set by a Wnt signaling gradient [36,37]. Irx transcription factors, as well as the zinc-finger transcription factors, FezF1 and FezF2, could be induced by specific levels of Wnt [38], thereby establishing a coarse prepattern of competence (Figure 2A).

Second, Shh signaling from the MDO induces a posterior-to-anterior expression wave of the proneural gene Neurogenin1 in the major (caudal) part of the thalamus, and Ascl1 (formerly Mash1) in the remaining narrow stripe of rostral thalamic cells immediately adjacent to the MDO, and in the prethalamus [39,40]. This zonation of proneural gene expression leads to the differentiation of glutamatergic relay neurons from the Neurogenin1-expressing precursors and of GABAergic inhibitory neurons from the Ascl1-positive precursors. In fish, selection of these alternative neurotransmitter fates is controlled by the dynamic expression of the hairy-like basic helix-loop-helix (bHLH) transcription factor, Her6. Expression of Her6, which represses Neurogenin but is required for Ascl1 expression, is progressively lost from the caudal thalamus but maintained in the prethalamus and in the stripe of rostral thalamic cells (Figure 2B). Studies in chick and mice have shown that blocking the Shh pathway leads to absence of the rostral thalamus and substantial decrease of the caudal thalamus [34,35]. Is a Shh concentration gradient in mouse solely responsible for defining the sharp border between rostral thalamus and caudal thalamus without need for a bHLH factor-mediated mechanism, as has been discovered in fish [40]? Recent reports support the hypothesis that these two requirements are possibly tightly linked, because Shh is able to influence the stability of the Her6 ortholog, Hes1, in mouse retina [41]. An increase of Shh signaling would lead to a broader domain of the stabilized bHLH factor, resulting in a broader rostral thalamus, whereas reduction of Shh signaling would reduce the activity of the bHLH factor and subsequently lead to the loss of rostral thalamic identity.

Because *her6* defines the border of the thalamic Neurogenin1 expression domain, it is pertinent to ask how the thalamus is limited posteriorly – from the largely GABAergic pretectum. It has been suggested that Otx2 is required for acquisition of glutamatergic fate in the thalamus, and that the absence of Otx2 leads to the thalamus taking on the GABAergic pretectal fate [42]. In zebrafish, normal expression of *gsh1* in the pretectum and the strong anterior expansion of *gsh1* expression in the embryos depleted for Otx function [11], suggests that the *Gsh* genes are required for the pretectal GABAergic fate. Indeed, *Gsh2* and the proneural bHLH gene, *Ascl1*, function sequentially to determine the identity of the dl3 interneurons in the mouse spinal cord: Gsh2 activates the Ascl1-dependent differentiation of these neurons by suppressing the expression of Neurogenin (1 and 2) [43,44]. Assuming a similar repressive interaction between Neurogenin1 in the posterior thalamus and *gsh1* in the anterior pretectum, pretectal cells could become GABAergic without the need for Her6.

**Figure 3:**
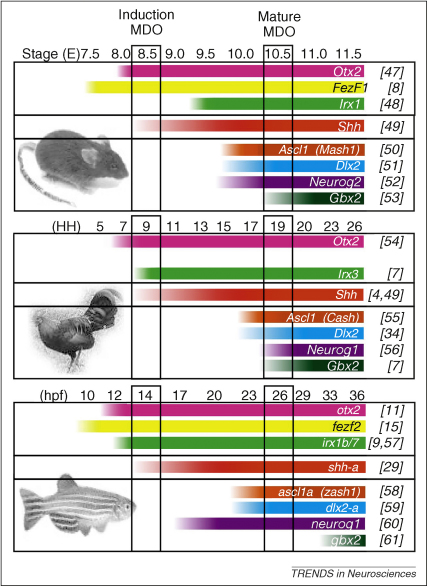
Comparative expression of thalamic markers in mouse, chick and zebrafish during embryonic development. Colored bars show the expression dynamics of genes expressed within the thalamic territory. Same colors indicate homologs or members of one gene family (such as FezF1 and FezF2). Expression dynamics in different model organisms have been staged according to two time points: induction of Shh in the MDO and its patterning activity from the mature MDO. Otx2, Fez, and Irx are involved in induction and positioning of the MDO, and Shh is the principal signaling molecule in the MDO. The prethalamic genes, Ascl1 and Dlx2, and the thalamic genes, neurogenin (neurog1 and 2) and gbx2, are target genes of Shh signaling. Fez expression has not yet been documented in chick. References to the relevant literature are given at the right hand site in square brackets.

A third Shh-dependent step involves the induction of a set of transcription factors that are likely to determine the finer specification of thalamic neuronal subtype identity (Figure 2C). The induction of these transcription factors occurs in a Shh-concentration-dependent manner (Figure 2C). At high levels of Shh signaling, Nkx2.2, Olig2, Sox14, Tal1 and Gad1 are induced in the Her6+ rostral thalamus, whereas the induction of Gbx2, Dbx1, Olig3, and Lhx2 in the caudal thalamus seems to require lower levels of Shh (Figure 3) [23,29,34,35,45,46]. Subsequently, the post-mitotic thalamic nuclei become reorganized by Shh: low activation of the Shh pathway within the thalamus leads to loss of the lateral geniculate and medial geniculate nucleus, and to a reduction of both the dorsal and the central nuclear groups [46]. These effects are consistent with Shh released from the MDO acting as a morphogen that patterns the AP axis of the diencephalon in a similar way to its actions along the dorsoventral axis of the spinal cord [62,63]. In addition, the duration of Shh exposure creates a graded response in the spinal cord similar to that generated by different Shh concentrations [64]. It will be interesting to explore the temporal effects of Shh exposure during diencephalic development, particularly because Shh expression is maintained in the ZLI and its derivative (the external medullary lamina) until post-natal stages.

It has recently been shown that one component of Shh-mediated nucleus formation is mediated via the activity of the transcription factor Gbx2. In mice lacking this Shh-dependent target gene, the central, medial, and posterior nuclear groups are not properly specified [46] and the lineage borders to the epithalamus and the pretectum are not maintained, resulting in the mixing of cell fates at these borders [65].

Wnt signaling is important for setting up the initial coarse AP regionalization of the neuraxis and it also has a more local role later in diencephalic development. Wnt ligands, receptors (Frizzled), and mediators (Lef1, Tcf7L2) are expressed in the mid-diencephalon, and recent detailed fine mapping of their expression suggests a complex pattern of Wnt activities in this region [66,67]. Wnt3 and Wnt3a are expressed in the dorsal region of the thalamus [68], whereas Wnt8b is expressed in the MDO itself [11,69] from the precursor stage onwards. Inhibition of canonical Wnt signaling by Wnt antagonists (eg. Dkk1) transforms thalamus into prethalamus, whereas ectopic Wnt signaling converts prethalamus into thalamus [70]. Furthermore, expression of Lhx5 in the prethalamus regulates the expression of the extracellular Wnt inhibitors Sfrp1a and Sfrp5 [71]. This is consistent with the observation that Wnt co-receptor LRP6 null mice have defective expression of thalamic markers, whereas prethalamic expression is roughly intact [72]. Future studies will show whether Wnt signaling is directly required for thalamic specification, or whether the above responses are referable to the overall Wnt-driven posteriorization of the neural tube [36,37].

In addition to Shh and Wnt signaling, FGF signaling has also been implicated in MDO function, although its specific role has yet to be clarified. Fgf15 and Fgf19 have been shown to act downstream of Shh in the thalamus and, therefore, are implicated in some aspects of thalamic development [73–75]. In zebrafish, Fgf3 and Fgf8 have been proposed to be involved in prethalamic development, because double morphants do not express the prethalamic marker *dlx2a* [76]. A recent report highlights a further possible role for Fgf8 in thalamic development [77], although the data could also be explained by an indirect epithalamus-mediated effect on thalamic development: the range of active Fgf8 signaling (as shown by Spry2 and Mkp3 target gene activation) is limited to regions close to the epithalamus and Fgf8 has a direct function on the development of the habenula and the pineal gland [78,79]. However, whereas the functions of these other signals have yet to be fully scrutinized, detailed studies in various model organisms have shown that Shh is the principal requirement for cell fate specification during thalamic development.

## Evolution of the MDO and the thalamic complex

**Figure 4:**
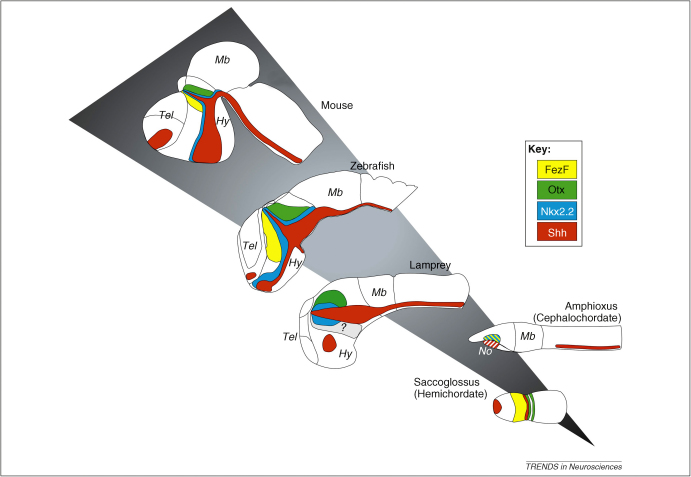
Evolution of the MDO. Expression of Shh (red) with the flanking expression domain of Nkx2.2 (blue), the prethalamic marker FezF (yellow) and the thalamic marker Otx (green) in the thalamic complex in agnathans (lamprey) and gnathostomes (zebrafish, mouse). Note that Shh is not expressed in the anterior neural tissue in Amphioxus (a cephalochordate) but in the underlying tip of the notochord (No). Data on Fez expression in lamprey are missing (grey). In Saccoglossus (a hemichordate) Shh is expressed in a narrow strip that lies between the Fez and Otx domain, just as in vertebrates, suggesting a basal chordate origin for the MDO. Expression summary in Amphioxus and lamprey is based on different, non-comparative publications. Except for Shh, expression domains are shown only for the thalamic complex. Hb, hindbrain; Hy, hypothalamus; Mb, midbrain; No, notochord; Pa, pallium, SPa, subpallium; Tel, telencephalon.

The diencephalon and its subregions can be visualized by the expression of sets of markers: Otx2 in the anterior neural plate, which later becomes restricted to the thalamic anlage, Fez and Lhx1/Lhx5 in the prethalamus, Shh in the MDO/ZLI and Lhx2/Lhx9 in the thalamus (Figure 4) [80,81]. Comparative molecular studies in non-vertebrate chordates have shown that the anterior neural ectoderm/ cerebral vesicle expresses an Otx homolog and could therefore correspond to the forebrain/midbrain of vertebrates [82–84]. In the urochordate *Ciona*, Shh (Ci-hh2) is expressed in the ventral nerve cord, but only at posterior levels, and is not expressed in the notochord [85]; in the cephalochordate *Amphioxus*, Shh (AmphiHh) is expressed in the notochord, which runs to the tip of the cerebral vesicle and, again, only in the posterior aspect of the ventral neural tube [86]. The cerebral vesicles of *Amphioxus* also express a homolog of the posterior prethalamic and rostral thalamic marker Nkx2.2, suggesting at least a similarity with the vertebrate diencephalon [87]. Remarkably, these expression domains lie close to the underlying Shh-positive notochord, suggesting that the anterior tip of the notochord could be the original mid-diencephalic organizer. A further parallel involves the Lim genes: AmphiLim1/5, the homolog of Lhx1/Lhx5, is expressed in the anterior neural ectoderm close to the Shh-positive notochord [88].

Analysis of lamprey forebrain gene expression indicates a high degree of similarity between agnathans and gnathostomes [89]. The expression of Shh in the interthalamic region of lamprey is suggestive of a functional MDO. How would the Shh source become translocated from the non-neural notochord of a cephalochordate ancestor into the agnathan neural tube? One possibility is that the notochord becomes physically separated from the neural tube early on in vertebrate development and to ensure consistency of signaling gradients, homeogenetic induction of a new Shh source was enforced within the basal plate and MDO [90]. Consistent with this notion, Shh expression in these structures is under the control of the same enhancer element, whereas floor plate and notochord expression share other regulatory elements [22,91], making it likely that forebrain Shh expression domains emerged separately from floor plate/notochord domains. Furthermore, the upstream mechanism inducing the MDO also seems to be conserved: lamprey *Otx* genes are first expressed in the whole anterior neural plate and subsequently become downregulated in the prethalamic anlage [92], as described for higher vertebrates. This leads to a new Otx border in the mid-diencephalon similar to that of gnathostomes, determining the future location of the MDO. Interestingly, the putative lamprey MDO is large, suggesting that the constraining mechanisms might not be in place or might be enforced later in development. This could be due to a lack of early Irx function, because knock-down of *irx1b* in zebrafish leads to a similarly broad MDO [11]. Thus, the MDO program might have been established progressively during evolution by the sequential recruitment of gene cascades.

It seems that even though the global AP order of regulatory gene expression is roughly conserved between cephalochordates and vertebrates, major differences in patterning mechanisms can be recognized. One of these is the lack of local organizers such as the MDO in cephalochordates, where the anterior tip of the notochord could have the organizing role. The transfer of CNS organizer properties from the axial mesoderm into the CNS itself can be seen as a key event in the evolution of the vertebrate brain.

However, this conclusion could be changed radically by recent studies of the hemichordate *Saccoglossus*. In the embryo of this protochordate, Otx is downregulated anteriorly to form a sharp expression border, and this appears close to an anterior domain of FezF, possibly setting up an interface similar that used in vertebrates to position the MDO. Furthermore, the homologs of Shh and Wnt8b are induced in a ring-like domain that aligns with the anterior Otx border. Further similarities with vertebrates include the expression of a Dlx homolog anterior to the Hh stripe and an Irx stripe posterior to it [84,93]. Although functional studies have yet to be done on these AP-patterning genes in *Saccoglossus*, their similarity in expression with vertebrates is astonishing. In comparison to this high degree of conservation, the morphology of Amphioxus and the molecular mechanisms required to develop it could have been specialized considerably by specific adaptation, and limit its usefulness in evolutionary studies. These recent findings highlight the importance of *Saccoglossus* as being closest to the central basic reference animal at the root of the chordate phylogenetic tree.

## Concluding remarks

Regionalization and compartition of the CNS has been studied for several decades, during which have we become aware of the orchestrating role of local organizers. Light has now been shed on the newly characterized organizer in mid-diencephalon and on several aspects of its formation, function and evolution. Areas for future research include a deeper understanding of how the MDO is positioned, and how its Shh signal is integrated with other signaling pathways. Given the established role of a Shh morphogen gradient in spinal cord patterning, the fact that this pathway plays an important role in the graduated specification of transcriptional codes within the thalamic complex is perhaps not surprising, but if – and how – it influences the differentiation of individual thalamic nuclei remains an open question. Rather striking is that an essential distinction between the thalamus and prethalamus exists as a pre-patterned difference in competence, requiring Shh signaling to evoke distinctions in gene expression profiles at a finer level of spatial pattern. A prepattern of Irx gene expression also dictates the distinct patterning of tectal versus cerebellar regionalization either side of the mid–hindbrain boundary [94]; thus, a crucial next step will be to study how the zonal expression of Irx factors in the neural plate is regulated – whether by vertical signals from the prechordal plate and notochord, as seems to be the case for mouse, or by a planar signal through the neuroectoderm – presumably involving interplay between Wnts and their inhibitors – as seen in zebrafish. Considering the multifunctional uses of the Shh signaling pathway and the expression of a number of other signal molecules at the MDO, much further research is needed into possible interactions between these signaling pathways before construction of the bridal chamber of the brain is fully understood.

## Box 1 Definition of a local organizer in development

The concept of ‘local organizers’ describes specific groups of cells that can direct development of the surrounding tissue by their release of signaling molecules. An organizer is characterized by the following features:

1. The signal molecule activity is produced by a defined (small) population of cells.
2. The ablation of this population of cells leads to the lack of surrounding structures.
3. The translocation of an organizer population to an ectopic competent location, such as by grafting, causes the induction of an ectopic structure.
4. Organizers refine simple spatial organization into a more complex spatial diversification of fields.
5. Ectopic provision of its principal signal is able to mimic the main functions of an organizer population.
6. The efficiency range of organizers depends on the timing of their appearance, on how their effective signaling molecules spread, and on how their signals are translated into specific responses at the target sites.

## Box 2 Function of an organizer: timing is everything.

Apparently independently of organizer activity, the neural tube shows an initial expression pattern of competence (modulating and determining) factors, anticipating the actual fate of subregions. The organizers define the timing of fate acquisition by the receiving tissue and the spatial extent of response, by initiating the appropriate downstream programs. Thus, the interplay between competent fields and organizer activity sets the stage for the next level of complexity during brain development.
